# Supplementary material for: Comparative analysis of milk and brain fatty acids reveals human-specific signatures in brain development
Source: Commun Biol. 2026 Apr 22;9:631. doi: 10.1038/s42003-025-09401-0 (PMC13161393; doi:10.1038/s42003-025-09401-0)
Supplement: Supplementary file 1 — Supplemental Material [file 42003_2025_9401_MOESM1_ESM.pdf]

Supplementary material

Supplementary figures

Supplementary Figure 1. Annotated FA in the milk dataset.
Supplementary Figure 2. Annotated FA in the brain dataset.
Supplementary Figure 3. Phylogenetic relationship.
Supplementary Figure 4. FA in milk across species and species groups.
Supplementary Figure 5. FAs in the brain across species and species groups.
Supplementary Figure 6. Factors contributing to the difference between the two human populations.
Supplementary Figure 7. Correlation between FAs in milk and in prefrontal cortex (PFC).
Supplementary Figure 8. Correlation between FAs in milk and in cerebellum (CB).
Supplementary Figure 9. FA intensity level changes in milk across lactation.
Supplementary Figure 10. FA intensity level changes in brain across age.

Supplementary tables

Supplementary Table 1. Human milk samples metadata.
Supplementary Table 2. Brain samples metadata.
Supplementary Table 3. Proportion of FAs in milk samples across all species.
Supplementary Table 4. Proportion of FAs in milk samples across phylogenetic groups.
Supplementary Table 5. Proportion of FAs in milk samples across primate species.
Supplementary Table 6. Proportion of FAs in milk samples across human populations.
Supplementary Table 7. Proportion of FAs in brain samples (prefrontal cortex) across all species.
Supplementary Table 8. Proportion of FAs in brain samples (prefrontal cortex) across primate species.
Supplementary Table 9. Proportion of FAs in brain samples (cerebellum) across all species.
Supplementary Table 10. Proportion of FAs in brain samples (cerebellum) across primate species.
Supplementary Table 11. Slope of the FAs change in brain samples (prefrontal cortex) across primate species.
Supplementary Table 12. Slope of the FAs change in brain samples (cerebellum) across primate species.
Supplementary Table 13. Correlation between the changes in milk and in brain.
Supplementary Table 14. List of annotated fatty acids in milk.
Supplementary Table 15. List of annotated fatty acids in brain.

Supplementary figures

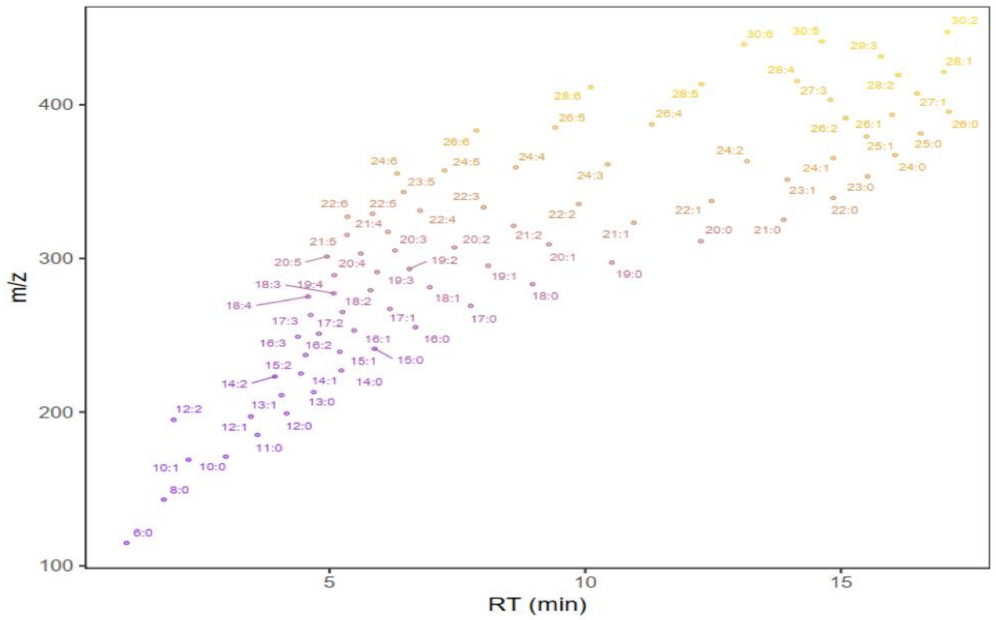

Supplementary Figure 1. Annotated FA in the milk dataset. 81 FA detected in milk plotted according to retention time (x-axis) and the mass-over-charge ratio (y-axis). FAs labelled by the length of the carbon chain and the number of the double bonds.

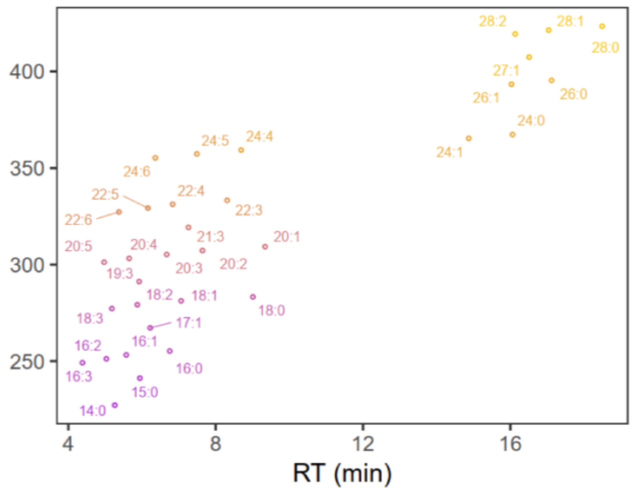

Supplementary Figure 2. Annotated FA in the brain dataset. 33 FA detected in brain plotted according to retention time (x-axis) and the mass-over-charge ratio (y-axis). FAs labelled by the length of the carbon chain and the number of the double bonds.

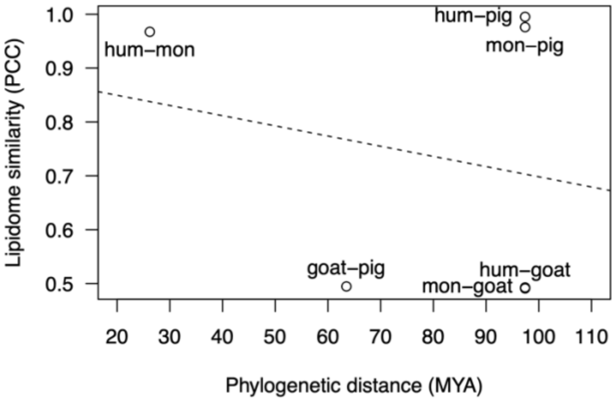

Supplementary Figure 3. Phylogenetic relationship. The relationship between the phylogenetic distances for each pair of species in million years ago (MYA) (x-axis) and lipid-intensity-based distances calculated as Pearson's correlation coefficient (PCC) between the lipid concentrations in milk for each pair of species. Dots are labelled by species pairs.

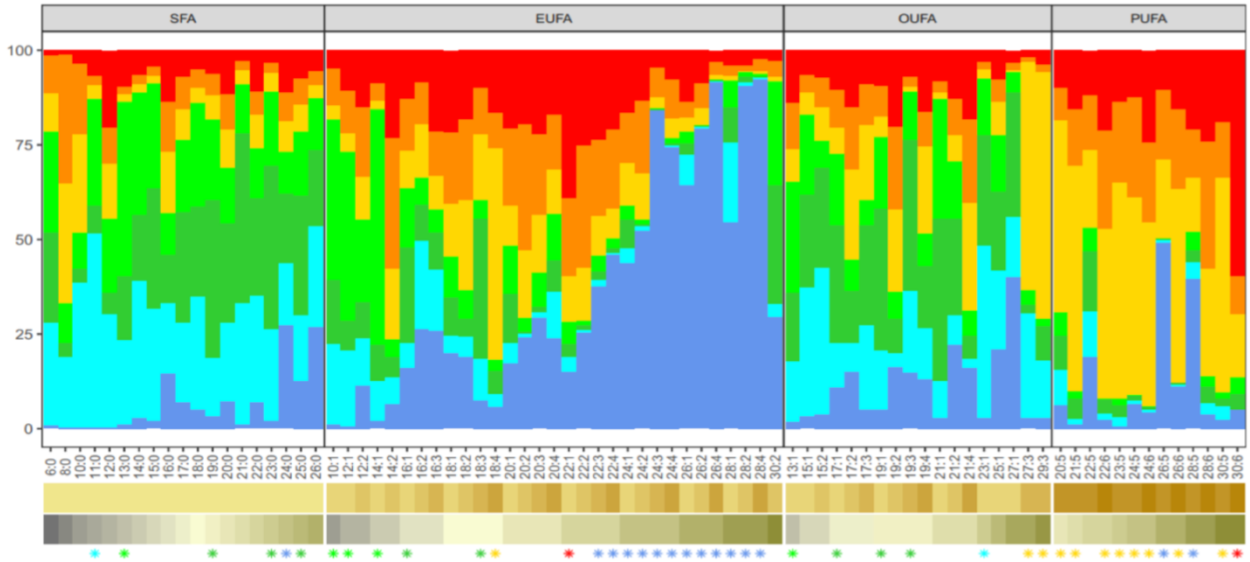

Supplementary Figure 4. FA in milk across species and species groups. Proportion of FA QIs (y-axis) across the seven species with 81 FAs classed into four groups (x-axis): short-chain FA (SFA), even-chain unsaturated FA (EUFA), odd-chain unsaturated FA (OUFA) and polyunsaturated FA (PUFA). FAs are presented in three colored bars along the x-axis: number of double bonds on the top, carbon chain length in the middle, chain parity (even or odd) on the bottom. Colored asterisks indicate species-specific FAs. FAs are labelled at the bottom.

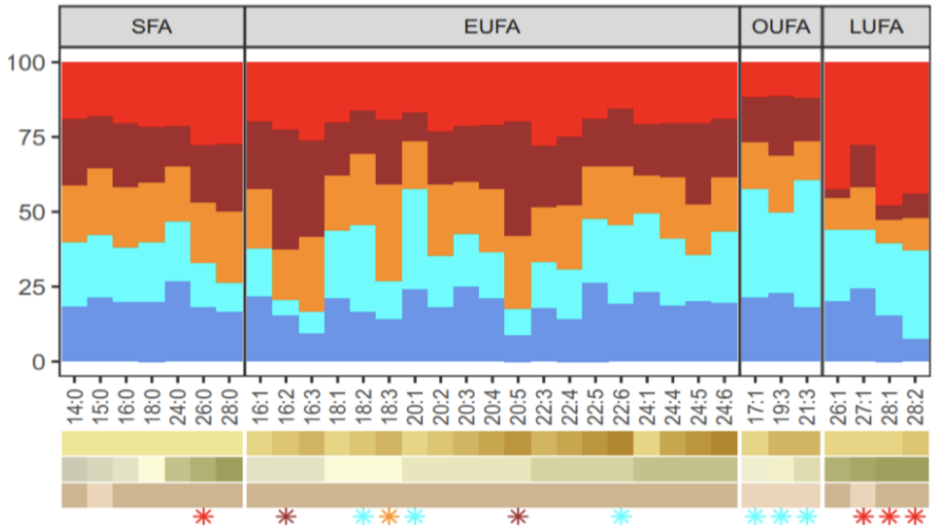

Supplementary Figure 5. FAs in the brain across species and species groups. Proportion of FA QIs (y-axis) across the seven species with 33 FAs classed into four groups (x-axis): short-chain FA (SFA), even-chain unsaturated FA (EUFA), odd-chain unsaturated FA (OUFA) and long-chain unsaturated FA (LUFA). FAs are presented in three colored bars along the x-axis: number of double bonds on the top, carbon chain length in the middle, chain parity (even or odd) on the bottom. Colored asterisks indicate species-specific FAs. FAs are labelled at the bottom.

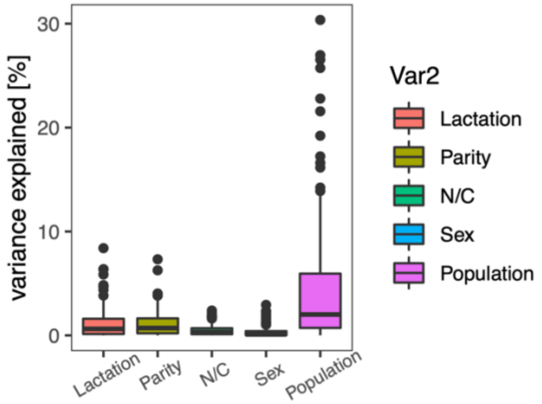

Supplementary Figure 6. Factors contributing to the difference between the two human populations. The percent of explained variance (y-axis) and five factors considered in the comparison of the lipid normalized intensity levels between the two human populations.

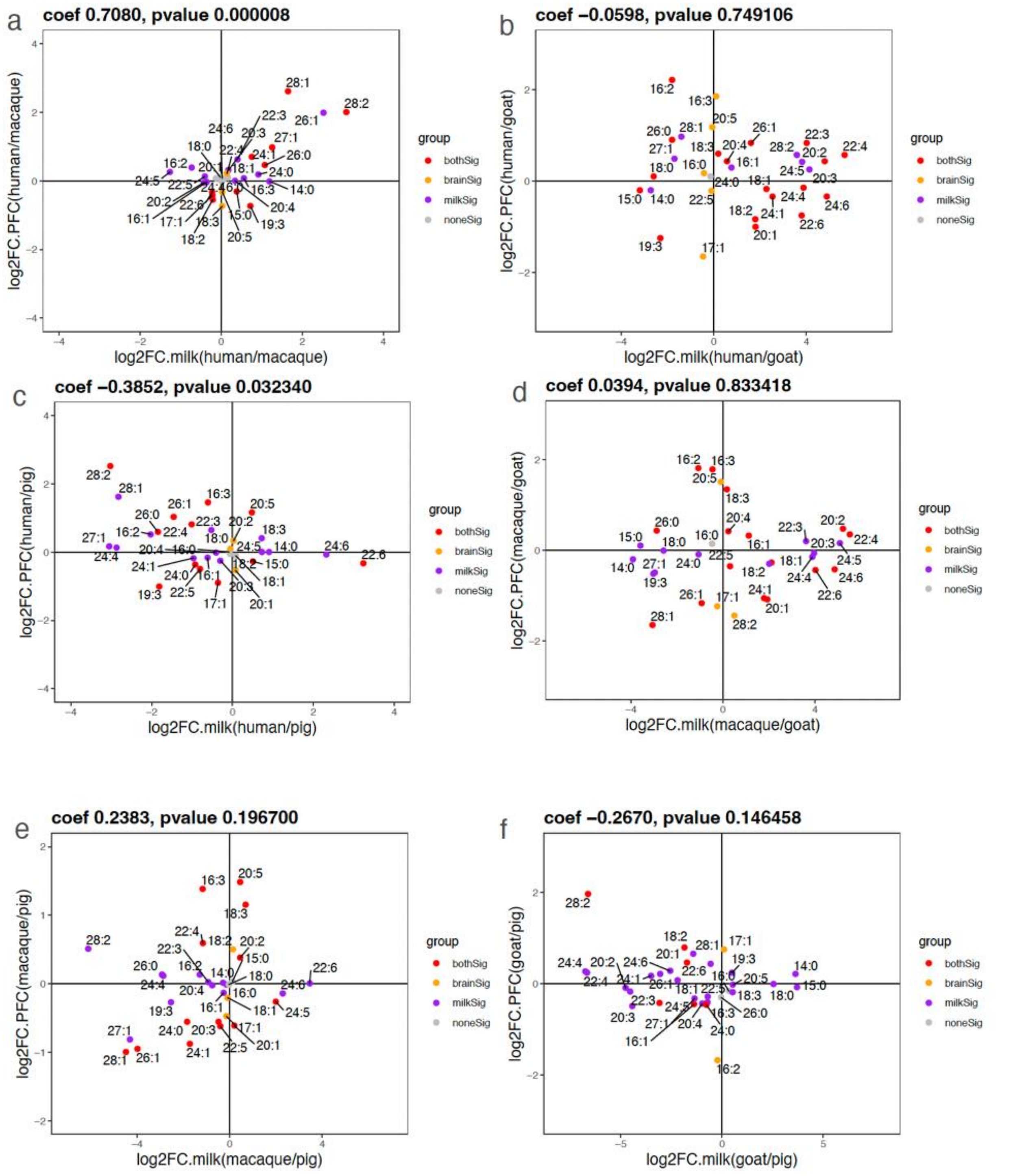

Supplementary Figure 7. Correlation between FAs in milk and in prefrontal cortex (PFC). Log2 fold change (logFC) in quantile-normalized FA intensities in milk (x-axis) and brain PFC (y-axis) between human and macaque (a), human and goat (b), human and pig (c), macaque and goat (d), macaque and pig (e) and goat and pig (f). Pearson's correlation coefficients and *p*-values are shown above each panel. Dots are labelled with the corresponding FAs.

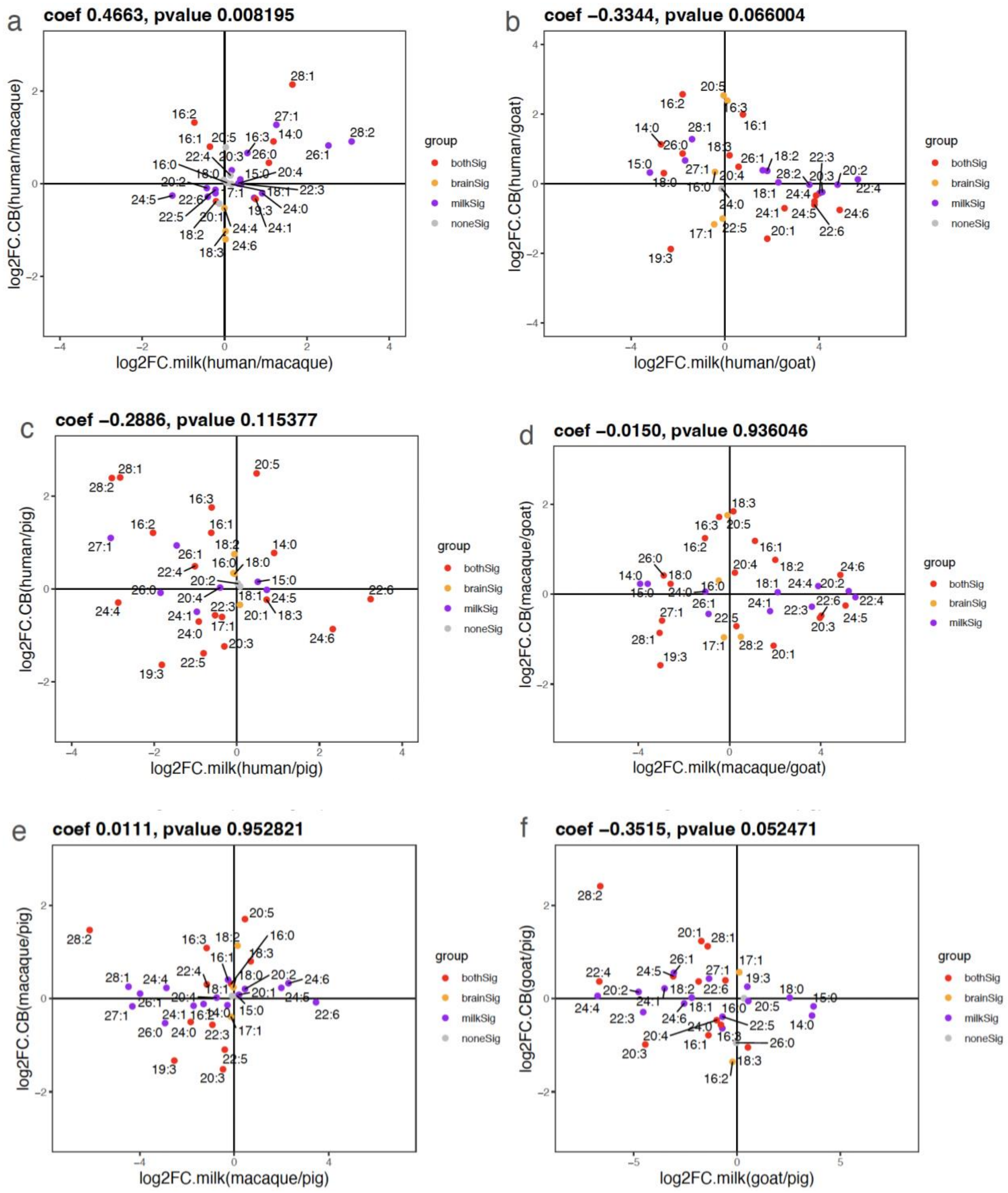

Supplementary Figure 8. Correlation between FAs in milk and in cerebellum (CB). Log2 fold change (logFC) in quantile-normalized FA intensities in milk (x-axis) and brain CB (y-axis) between human and macaque (a), human and goat (b), human and pig (c), macaque and goat (d),

macaque and pig (e) and goat and pig (f). Pearson's correlation coefficients and *p*-values are shown above each panel. Dots are labelled with the corresponding FAs.

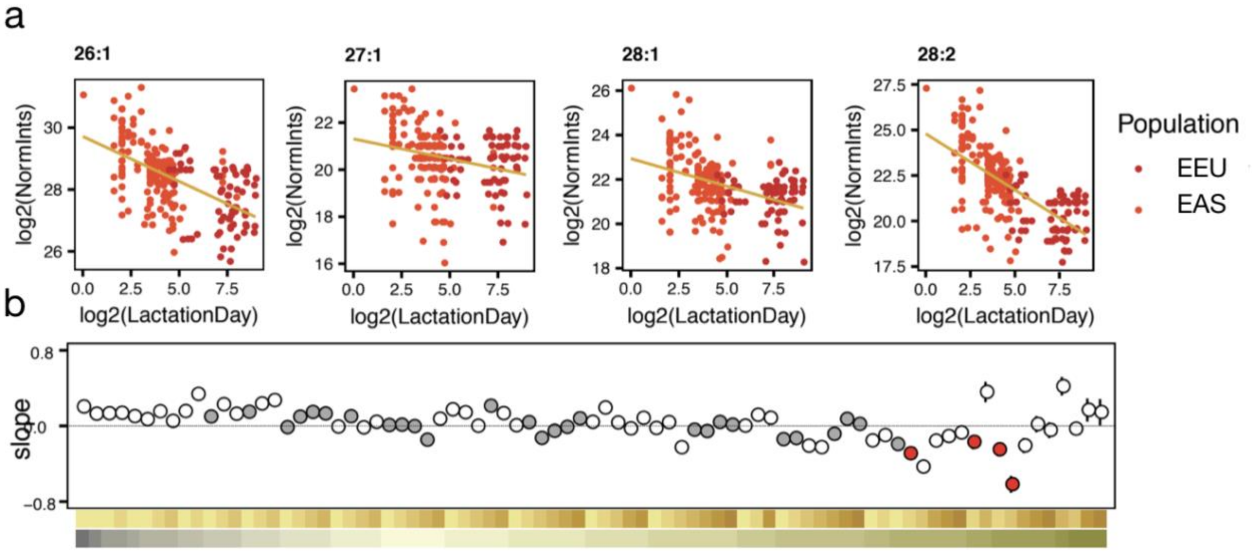

Supplementary Figure 9. FA intensity level changes in milk across lactation. **a** Normalized intensity changes (y-axis) in 4 FAs across lactation (x-axis) in milk of individuals representing Eastern European (dark red) and East Asian (red) human populations. **b** Normalized intensity changes of the 81 FAs (x-axis) detected and annotated in milk and shown here as a slope of the change (y-axis) in the milk of samples from human participants.

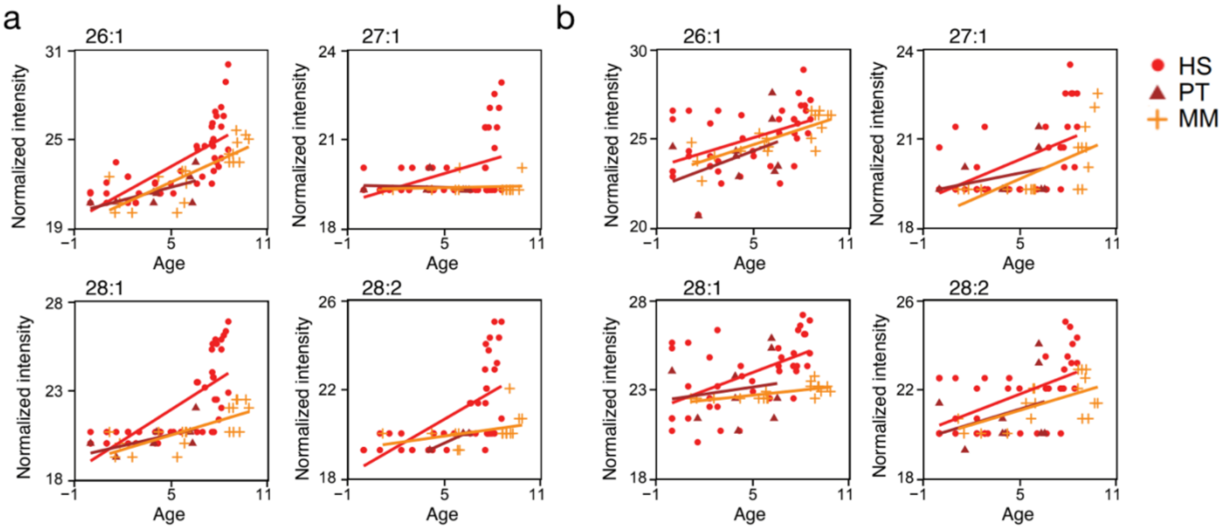

Supplementary Figure 10. FA intensity level changes in brain across age. Normalized intensity level (y-axis) of the four FAs from the LUFA group in the brain PFC (a) and CB (b) across age (x-axis) in humans (HS), chimpanzees (PT) and macaques (MM).

Supplementary tables

Supplementary Table 1. Human milk samples metadata.

	Total number of samples	Parity					Parturition			Baby's Sex		
		1st	2nd	3rd	4th	undef.	N	C	undef.	F	M	undef.
Moscow cohort	297	50	71	14	6	156	142	68	87	136	74	87
Shanghai cohort	291	167	91	0	0	33	106	155	30	140	126	25

	Baby's height at birth (cm)	Baby's weight at birth (g)	Mother's mean age (years)	Mother's mean height (cm)	Mother's mean weight (kg)
Moscow cohort	51	3300	31	166	60
Shanghai cohort	51	3200	32	165	63

Supplementary Table 2. Brain samples metadata.

	Total number of samples	Region		Age (days)			Sex	
		PFC	CB	min	max	med	F	M
**Human**	92	43	49	0	365	30	52	40
**Chimpanzee**	18	9	9	0	45	7.5	8	10
**Macaque**	38	19	19	0	358	24	2	36
**Pig**	20	10	10	-	-	-	-	-
**Goat**	26	13	13	5	86	45	14	12

Supplementary Table 3. Proportion of FAs in milk samples across all species.

FA	FA.group	HS	MM	MF	BT	BG	CH	SS
6:0	SFA	1.29632423	10.016624	10.106574	26.7248316	23.6930244	27.457795	0.70482682
8:0	SFA	1.09210196	33.8982213	31.7689224	10.5112108	3.72666938	18.6463462	0.35652789
10:0	SFA	3.63474382	18.5907752	25.9751507	9.51498013	3.64075841	38.3482208	0.29537097
11:0	SFA	6.8218405	2.39949082	3.60465172	28.0861823	7.37435154	51.4511145	0.26236868
12:0	SFA	20.3507045	9.74123476	14.2367724	19.8064887	5.40395962	30.0431089	0.41773116
13:0	SFA	9.6936552	1.92727491	1.80337787	46.1224009	17.1018411	22.3083774	1.0430726
14:0	SFA	6.53661575	2.3474845	2.10400075	32.5131358	17.4027474	36.2437782	2.85223751
15:0	SFA	4.26661973	2.40846909	1.84380187	27.8852161	31.8521018	29.5384417	2.20534964
16:0	SFA	13.5396428	13.2416714	16.0962274	11.1744489	12.6273006	18.8417856	14.4789232
17:0	SFA	7.07229427	8.44641853	7.93828241	19.2708535	29.0966208	21.2523074	6.92322315
18:0	SFA	4.92078818	4.89785425	4.11471143	27.4388414	23.6918888	29.950228	4.98568795
19:0	SFA	6.27815485	5.44379094	6.53299834	21.153182	41.79794	15.5188294	3.27510441
20:0	SFA	11.7371805	9.16549365	10.115961	14.5475683	26.338053	20.8763874	7.21935623
21:0	SFA	2.78777044	2.44252709	3.65057263	12.878707	45.0850414	31.930471	1.22491045
22:0	SFA	11.0026855	5.90532147	8.93136839	13.0637724	25.9506139	28.048839	7.09739927
23:0	SFA	3.38713004	2.55544673	4.79342293	19.8395564	43.1424959	24.1205931	2.16135491
24:0	SFA	11.0400628	7.70072741	7.96538943	11.1299837	18.3107925	16.4551841	27.3978601
25:0	SFA	7.35831505	6.90606735	7.1617873	16.7956097	31.6403574	17.5979141	12.5399491
26:0	SFA	5.47403385	3.54703838	3.48699542	13.8738602	20.0460357	26.7550579	26.8169785
10:1	EUFA	4.74484918	9.6813555	3.84235352	42.3906013	16.8943295	21.1791233	1.2673877
12:1	EUFA	10.7040389	11.1882343	4.88158122	44.5251631	7.94382991	20.1485541	0.60859852
12:2	EUFA	14.9991133	18.3338678	11.3061227	21.89222	9.61849417	12.4161378	11.4340443
14:1	EUFA	8.79987009	4.62528536	2.07666612	62.3705693	9.32672876	10.6459208	2.15495956
14:2	EUFA	23.2330142	34.3340826	18.7380788	4.73877096	5.27190697	7.1860833	6.49806312
16:1	EUFA	12.9343025	13.5797328	9.96969697	15.6586713	25.2219503	6.40729898	16.2283472
16:2	EUFA	8.48796673	10.8001144	14.3061876	7.09346537	9.67384742	23.2463281	26.3920904
16:3	EUFA	21.51612	11.5884067	9.01294441	6.12026117	9.63999718	16.2245262	25.8977443
18:1	EUFA	21.5796894	18.9634544	13.9534658	10.7720501	10.1671229	4.51010417	20.0541132
18:2	EUFA	18.1772117	21.2781166	23.8689185	7.61552725	4.50964758	5.4271006	19.1234777
18:3	EUFA	9.85558957	12.1655295	17.5428249	4.76193811	37.122186	11.0744583	7.47747359
18:4	EUFA	16.4284132	9.38788196	55.9456346	2.89830751	6.05427926	3.59531729	5.69016617
20:1	EUFA	20.778125	20.1343314	10.6427533	12.8391479	12.7630513	5.41666993	17.4259213
20:2	EUFA	19.4389364	33.2461112	18.0769676	3.51374754	0.63861331	0.91582023	24.1698038
20:3	EUFA	22.2344452	21.2334938	15.2272594	9.1072441	1.37931144	1.39762417	29.4206219
20:4	EUFA	17.0104534	14.3178956	11.8257656	12.3556088	8.1481259	12.4641053	23.8780455
22:1	EUFA	39.1258107	20.4441357	12.2055271	5.65756162	3.59836092	3.91435627	15.0542478
22:2	EUFA	25.169982	32.2246151	14.1463022	1.14339993	1.09040992	0.97708847	25.2482024
22:3	EUFA	23.7588472	19.8832811	10.6989098	3.97536449	2.42392903	1.66876513	37.5909033
22:4	EUFA	20.8965963	20.7551905	7.93232221	2.73734112	1.23055263	0.46665338	45.9813439
24:1	EUFA	16.4266874	13.365381	11.2522415	3.70004599	7.36949891	3.99058327	43.895562
24:2	EUFA	13.2825131	19.1938299	12.0795823	1.3272819	0.63116844	1.04376175	52.4418626

24:3	EUFA	4.63586181	7.69957366	2.85209038	0.4024743	0.10707	0.08764017	84.2152897
24:4	EUFA	7.66171593	10.2430143	5.2727983	1.42879463	0.37437723	0.71243234	74.3068673
26:1	EUFA	13.6790452	4.10300873	3.50999631	3.24559995	3.06335442	7.95246333	64.4465321
26:2	EUFA	8.77174289	6.52392558	4.27988839	0.47721173	0.18428867	0.34119068	79.4217521
26:4	EUFA	2.93318648	3.50858663	1.22811008	0.23494774	0.19763219	0.3142243	91.5833126
28:1	EUFA	4.12344965	2.46694918	1.44506272	7.01836903	9.29046353	21.0227601	54.6329458
28:2	EUFA	4.01967625	1.31524906	0.2523919	1.37712412	1.431094	0.95413975	90.6503249
28:4	EUFA	2.34186902	3.07183698	0.79424731	0.80505667	0	0.70610272	92.2808873
30:2	EUFA	2.8869088	3.9843247	1.2873769	27.3478712	31.4934121	3.32065751	29.6794488
13:1	OUFA	13.8159406	12.097899	8.72034516	29.4142604	18.1564781	16.0177978	1.77727902
15:1	OUFA	6.60786475	4.50647672	5.92063763	20.9071575	24.6981302	33.9457404	3.41399284
15:2	OUFA	7.29201091	7.67442874	8.7998239	7.33443353	26.4054699	38.5294176	3.96441534
17:1	OUFA	10.48717	9.90477721	6.95677749	18.6427241	31.154816	11.9999742	10.853761
17:2	OUFA	15.0761131	16.3608058	23.8355732	8.12395168	13.927662	7.64965407	15.0262402
17:3	OUFA	8.91871082	10.6841821	19.9514535	6.504365	26.5285127	22.4527088	4.96006702
19:1	OUFA	9.42718825	7.89873642	5.40208047	19.0810469	37.425562	15.7733659	4.9920201
19:2	OUFA	20.1979355	21.7789828	21.9549394	7.00397597	8.95901148	3.88812657	16.2170283
19:3	OUFA	6.91626643	2.59161267	1.32701775	12.8486461	39.739018	21.6523069	14.9251321
19:4	OUFA	16.2987338	8.86713372	23.2536178	8.42306519	16.4404996	13.6633527	13.0535973
21:1	OUFA	8.30514762	2.70883399	1.73192992	31.7694692	42.875685	9.64415347	2.96478074
21:2	OUFA	12.7922081	9.62729143	6.85063088	15.2099884	25.4184613	7.90389721	22.1975225
21:4	OUFA	18.2099309	21.9643927	28.5260506	6.75249423	6.04079242	2.50730133	15.9990378
23:1	OUFA	3.10231309	1.72765508	2.60464118	14.993394	29.0991928	45.7188214	2.7539825
25:1	OUFA	7.64089983	5.93458617	8.65518334	15.0547136	21.043898	20.8214049	20.8493142
27:1	OUFA	3.18756134	2.02308801	0.54692569	5.34789158	32.8140679	16.100907	39.9795586
27:3	OUFA	1.84378933	1.16688624	60.2840693	3.89047006	2.3843319	27.4903909	2.94006223
29:3	OUFA	3.84993052	1.79055357	65.3314049	1.83700787	9.21421413	15.1820119	2.79487709
20:5	PUFA	9.95945687	8.54233296	50.6337536	6.29120572	9.05608798	9.30069514	6.21646776
21:5	PUFA	15.4698332	15.099649	59.4587969	2.0734379	5.32267068	1.5164031	1.05920928
22:5	PUFA	11.7587358	14.4733801	20.7825656	5.98574584	15.9662785	11.9978113	19.0354828
22:6	PUFA	21.2926412	25.9586171	44.7101994	0.39490577	3.65718377	1.63862441	2.34782836
23:5	PUFA	13.6789329	21.3029561	57.0555424	1.55648221	3.3713117	2.25819897	0.77657569
24:5	PUFA	12.3111503	26.5239579	52.0298087	0.32260556	1.37185807	0.80427053	6.63634899
24:6	PUFA	24.2595848	21.2940041	48.5070476	0.19079979	0.68416057	0.75402107	4.31038198
26:5	PUFA	10.3244705	18.5389657	20.7129224	0.14786644	0.25556017	0.78744132	49.2327734
26:6	PUFA	15.5861834	21.0182227	51.1500049	0.19249597	0.39752954	0.54249335	11.1130702
28:5	PUFA	20.9413554	12.6288641	14.3802663	4.68984627	3.41488429	4.23369814	39.7110854
28:6	PUFA	24.0837328	33.4764177	28.6088615	2.50566791	4.57850321	2.97279676	3.77402024
30:5	PUFA	19.043075	14.6701058	56.5226006	1.93537076	1.91037365	3.44983216	2.46864202
30:6	PUFA	59.7521487	9.95730763	16.6749616	4.33526976	4.27927573	0	5.00103653

Abbreviations: HS, Homo sapiens; MM, Macaca mulatta; MF, Macaca fascicularis; BT, Bos taurus; BG, Bos grunniens; CH, Capra hircus; SS, Sus scrofa; SFA, short-chain fatty acids; EUFA, even-chain unsaturated fatty acids; OUFA, odd-chain unsaturated fatty acids; PUFA, polyunsaturated fatty acids.

Supplementary Table 4. Proportion of FAs in milk samples across phylogenetic groups.

FA	FA.group	PR	BO	CH	SS
6:0	SFA	3.33397047	46.707684	48.7080365	1.25030909
8:0	SFA	10.0494608	30.2486227	58.5818017	1.12011469
10:0	SFA	9.35271638	16.736119	73.3462272	0.56493746
11:0	SFA	7.85223955	30.3440107	61.4901881	0.31356171
12:0	SFA	29.0393873	26.2941298	44.0539396	0.61254323
13:0	SFA	12.2518079	56.5583595	29.7966317	1.39320084
14:0	SFA	8.23467976	40.2496709	47.7573413	3.75830797
15:0	SFA	6.41545208	44.1921046	45.9609815	3.43146179
16:0	SFA	23.3609255	19.4899377	32.3159927	24.8331441
17:0	SFA	12.7896666	36.7809159	38.0380231	12.3913944
18:0	SFA	7.32513606	40.3588329	44.8500351	7.46599592
19:0	SFA	12.8050074	48.758209	31.7386394	6.69814425
20:0	SFA	20.7962193	28.8329934	37.4277358	12.9430515
21:0	SFA	5.2936496	32.189219	60.2074645	2.30966692
22:0	SFA	17.7560355	24.3023792	46.2409145	11.7006708
23:0	SFA	6.48861084	43.5132163	45.886461	4.11171181
24:0	SFA	16.2218817	18.0758726	24.6537628	41.0484828
25:0	SFA	13.0548784	33.3220678	31.311241	22.3118129
26:0	SFA	7.26121837	19.9438958	36.3553734	36.4395125
10:1	EUFA	7.33160306	58.8558489	31.9034047	1.90914337
12:1	EUFA	14.8009333	55.9775972	28.3646977	0.85677181
12:2	EUFA	25.327319	34.3289971	21.0024703	19.3412136
14:1	EUFA	10.9940728	72.3160799	13.880201	2.80964627
14:2	EUFA	55.8795162	11.4717303	17.1451441	15.5036094
16:1	EUFA	24.5276853	32.265501	12.2302218	30.9765919
16:2	EUFA	13.314627	11.2851383	35.3109274	40.0893072
16:3	EUFA	29.8924288	9.4700701	23.3561656	37.2813356
18:1	EUFA	37.5822984	18.9307486	7.98440614	35.5025469
18:2	EUFA	36.7756447	14.3544833	10.8030739	38.0667981
18:3	EUFA	27.0347146	23.7890652	29.3554333	19.8207869
18:4	EUFA	58.2928166	10.9582508	11.9059141	18.8430184
20:1	EUFA	36.3823071	22.8798631	9.66017275	31.077657
20:2	EUFA	41.255192	6.53416661	1.90609416	50.3045472
20:3	EUFA	36.0669731	13.3079003	2.29587694	48.3292496
20:4	EUFA	25.8014137	18.1957994	19.2070259	36.7957609
22:1	EUFA	60.6815066	8.69964945	6.31849678	24.3003472
22:2	EUFA	47.7459093	2.17043005	1.86599141	48.2176693
22:3	EUFA	34.9982515	5.71795929	2.51990718	56.763882
22:4	EUFA	29.4346625	3.66039834	0.67218002	66.2327591
24:1	EUFA	23.6738772	6.12458615	5.85023237	64.3513043
24:2	EUFA	19.6994836	1.81470277	1.53163568	76.954178

24:3	EUFA	5.22586096	0.40747423	0.09810229	94.2685625
24:4	EUFA	9.12003692	1.53810975	0.84844867	88.4934047
26:1	EUFA	14.6897001	3.63469923	8.97142586	72.7041748
26:2	EUFA	9.62651606	0.49476494	0.38446151	89.4942575
26:4	EUFA	3.04253924	0.24210577	0.33069782	96.3846572
28:1	EUFA	4.57430257	8.41217961	24.1788017	62.8347161
28:2	EUFA	3.92798142	1.43004646	0.98577801	93.6561941
28:4	EUFA	2.40305877	0.83631907	0.73475804	96.0258641
30:2	EUFA	4.50334779	43.7390175	5.20814621	46.5494886
13:1	OUFA	22.8945429	47.110201	26.9994871	2.99576901
15:1	OUFA	9.98494496	32.7836681	52.0014903	5.2298967
15:2	OUFA	12.3301366	16.45279	64.572955	6.64411843
17:1	OUFA	19.3463033	37.9121904	22.4425884	20.2989179
17:2	OUFA	32.8333133	18.9002509	16.2825568	31.9838789
17:3	OUFA	20.4047867	19.8554433	48.9304575	10.8093126
19:1	OUFA	17.946548	41.7103082	30.6446107	9.69853318
19:2	OUFA	42.6012949	15.2255802	8.15584107	34.0172839
19:3	OUFA	11.0636166	27.4726883	36.3839248	25.0797704
19:4	OUFA	31.0732318	18.0333343	26.0274821	24.8659518
21:1	OUFA	14.7023422	61.8272419	17.9517388	5.51867715
21:2	OUFA	21.1144774	27.9732187	13.3683268	37.5439771
21:4	OUFA	42.6180756	15.1852471	5.71694834	36.4797289
23:1	OUFA	4.4507559	24.6248101	66.894862	4.02957193
25:1	OUFA	11.7051998	24.3125002	31.9697236	32.0125763
27:1	OUFA	4.49234391	13.1107758	23.656446	58.7404343
27:3	OUFA	11.6126148	9.48024769	71.2834622	7.62367531
29:3	OUFA	23.4819727	11.0531725	55.2869969	10.177858
20:5	PUFA	33.8631589	19.8429695	27.7476746	18.546197
21:5	PUFA	77.031547	11.3049066	6.86696419	4.79658223
22:5	PUFA	24.0924272	14.4313824	23.7673684	37.708822
22:6	PUFA	82.2463438	3.02756338	6.0531346	8.6729582
23:5	PUFA	76.1899732	8.84203918	11.1377938	3.83019387
24:5	PUFA	64.2103039	2.04070121	3.64799222	30.1010027
24:6	PUFA	82.4684753	0.84049762	2.48506804	14.2059591
26:5	PUFA	17.9085152	0.2655143	1.28814221	80.5378283
26:6	PUFA	58.7530727	0.98994924	1.87370974	38.3832684
28:5	PUFA	29.67366	6.65640691	6.13404493	57.5358881
28:6	PUFA	71.3502052	9.03908974	8.6409103	10.9697948
30:5	PUFA	72.8064812	6.69262279	11.9498114	8.55108457
30:6	PUFA	85.6716595	6.64729971	0	7.68104082

Abbreviations: *PR, primates; BO, bovines; CH, Capra hircus; SS, Sus scrofa; SFA, short-chain fatty acids; EUFA, even-chain unsaturated fatty acids; OUFA, odd-chain unsaturated fatty acids; PUFA, polyunsaturated fatty acids.*

Supplementary Table 5. Proportion of FAs in milk samples across primate species.

FA	FA.group	HS	MM	MF
6:0	SFA	6.05206887	46.7639937	47.1839374
8:0	SFA	1.63588122	50.776819	47.5872998
10:0	SFA	7.54085751	38.5695372	53.8896053
11:0	SFA	53.187662	18.7080461	28.104292
12:0	SFA	45.9086307	21.9750008	32.1163685
13:0	SFA	72.20972	14.3566053	13.4336748
14:0	SFA	59.4881295	21.3638781	19.1479924
15:0	SFA	50.0842173	28.2720976	21.6436851
16:0	SFA	31.57747	30.8825341	37.539996
17:0	SFA	30.1500436	36.008101	33.8418554
18:0	SFA	35.3166096	35.1520122	29.5313783
19:0	SFA	34.3915314	29.8209126	35.7875559
20:0	SFA	37.8391262	29.548346	32.6125278
21:0	SFA	31.3907353	27.5032406	41.1060241
22:0	SFA	42.5810815	22.8539638	34.5649547
23:0	SFA	31.5492747	23.8025968	44.6481284
24:0	SFA	41.338982	28.8350019	29.8260161
25:0	SFA	34.3426527	32.2319269	33.4254204
26:0	SFA	43.764025	28.3580044	27.8779706
10:1	EUFA	25.9727622	52.9946337	21.0326041
12:1	EUFA	39.9794467	41.7879105	18.2326427
12:2	EUFA	33.6008388	41.071317	25.3278442
14:1	EUFA	56.7666842	29.8370443	13.3962716
14:2	EUFA	30.4474946	44.9957455	24.5567599
16:1	EUFA	35.452246	37.2213366	27.3264174
16:2	EUFA	25.2661155	32.1486813	42.5852032
16:3	EUFA	51.0859732	27.5144884	21.3995384
18:1	EUFA	39.5982238	34.7974938	25.6042824
18:2	EUFA	28.7049789	33.6018472	37.6931739
18:3	EUFA	24.9105336	30.7490313	44.3404351
18:4	EUFA	20.0929861	11.4819721	68.4250418
20:1	EUFA	40.3026681	39.053922	20.6434099
20:2	EUFA	27.4708632	46.982991	25.5461458
20:3	EUFA	37.8811995	36.1758618	25.9429388
20:4	EUFA	39.4179179	33.1785177	27.4035644
22:1	EUFA	54.5113934	28.4834564	17.0051502
22:2	EUFA	35.1826469	45.043626	19.7737271
22:3	EUFA	43.7217396	36.5898073	19.6884531
22:4	EUFA	42.1437367	41.8585528	15.9977105
24:1	EUFA	40.0218385	32.5632981	27.4148634
24:2	EUFA	29.8108794	43.0780637	27.1110569

24:3	EUFA	30.524141	50.696695	18.7791639
24:4	EUFA	33.0566562	44.1937297	22.7496141
26:1	EUFA	64.2448475	19.270144	16.4850086
26:2	EUFA	44.8096724	33.3268965	21.8634311
26:4	EUFA	38.2429094	45.7449813	16.0121093
28:1	EUFA	51.315654	30.7007776	17.9835684
28:2	EUFA	71.9428681	23.5399031	4.51722875
28:4	EUFA	37.7236893	49.4822823	12.7940284
30:2	EUFA	35.3848101	48.835825	15.7793649
13:1	OUFA	39.8910518	34.9305147	25.1784334
15:1	OUFA	38.7899786	26.4542545	34.7557669
15:2	OUFA	30.6821932	32.2912717	37.026535
17:1	OUFA	38.3461025	36.2165963	25.4373013
17:2	OUFA	27.2759786	29.600268	43.1237534
17:3	OUFA	22.5479919	27.0113984	50.4406097
19:1	OUFA	41.4782916	34.7533203	23.7683881
19:2	OUFA	31.5929119	34.0659314	34.3411567
19:3	OUFA	63.8332467	23.9191264	12.247627
19:4	OUFA	33.661518	18.3131516	48.0253304
21:1	OUFA	65.159307	21.2525718	13.5881213
21:2	OUFA	43.703967	32.8911805	23.4048526
21:4	OUFA	26.5063052	31.9712854	41.5224094
23:1	OUFA	41.7279906	23.2380076	35.0340019
25:1	OUFA	34.3709841	26.6954903	38.9335256
27:1	OUFA	55.3629144	35.1378488	9.49923684
27:3	OUFA	2.91302119	1.84357523	95.2434036
29:3	OUFA	5.42458511	2.52290533	92.0525096
20:5	PUFA	14.405697	12.3559208	73.2383823
21:5	PUFA	17.1833043	16.7721178	66.0445779
22:5	PUFA	25.0107742	30.7848094	44.2044164
22:6	PUFA	23.1538753	28.2277138	48.618411
23:5	PUFA	14.8623583	23.1459699	61.9916718
24:5	PUFA	13.5488489	29.1905378	57.2606133
24:6	PUFA	25.7914317	22.6385924	51.5699759
26:5	PUFA	20.825391	37.3947708	41.7798382
26:6	PUFA	17.7611395	23.9511866	58.2876739
28:5	PUFA	43.6728743	26.3373017	29.989824
28:6	PUFA	27.9494127	38.8497175	33.2008698
30:5	PUFA	21.103685	16.2575262	62.6387888
30:6	PUFA	69.1700542	11.5267404	19.3032054

Abbreviations: HS, Homo sapiens; MM, Macaca mulatta; MF, Macaca fascicularis; SFA, short-chain fatty acids; EUFA, even-chain unsaturated fatty acids; OUFA, odd-chain unsaturated fatty acids; PUFA, polyunsaturated fatty acids.

Supplementary Table 6. Proportion of FAs in milk samples across human populations.

FA	FA.group	HSM	HSS
6:0	SFA	51.6678093	48.3321907
8:0	SFA	48.814197	51.185803
10:0	SFA	46.242008	53.757992
11:0	SFA	51.0456537	48.9543463
12:0	SFA	48.1998019	51.8001981
13:0	SFA	58.2666084	41.7333916
14:0	SFA	51.8572908	48.1427092
15:0	SFA	57.2968463	42.7031537
16:0	SFA	50.0089864	49.9910136
17:0	SFA	46.846926	53.153074
18:0	SFA	49.0000696	50.9999304
19:0	SFA	59.3264629	40.6735371
20:0	SFA	46.3223698	53.6776302
21:0	SFA	48.4348129	51.5651871
22:0	SFA	41.2085532	58.7914468
23:0	SFA	50.4496563	49.5503437
24:0	SFA	41.0691591	58.9308409
25:0	SFA	45.1657631	54.8342369
26:0	SFA	44.962691	55.037309
10:1	EUFA	48.1690094	51.8309906
12:1	EUFA	49.404064	50.595936
12:2	EUFA	43.0826117	56.9173883
14:1	EUFA	54.1835602	45.8164398
14:2	EUFA	50.5801381	49.4198619
16:1	EUFA	49.7808715	50.2191285
16:2	EUFA	47.5835358	52.4164642
16:3	EUFA	41.0009596	58.9990404
18:1	EUFA	50.5173014	49.4826986
18:2	EUFA	48.120766	51.879234
18:3	EUFA	46.7366318	53.2633682
18:4	EUFA	41.3442093	58.6557907
20:1	EUFA	57.7979936	42.2020064
20:2	EUFA	47.5995091	52.4004909
20:3	EUFA	53.3841874	46.6158126
20:4	EUFA	42.3075731	57.6924269
22:1	EUFA	67.2845093	32.7154907
22:2	EUFA	52.2379465	47.7620535
22:3	EUFA	57.7845077	42.2154923
22:4	EUFA	47.2840842	52.7159158
24:1	EUFA	59.2664915	40.7335085
24:2	EUFA	57.6850229	42.3149771

24:3	EUFA	61.2187982	38.7812018
24:4	EUFA	42.013757	57.986243
26:1	EUFA	58.8801244	41.1198756
26:2	EUFA	54.6989177	45.3010823
26:4	EUFA	43.8230547	56.1769453
28:1	EUFA	50.5328195	49.4671805
28:2	EUFA	42.0008909	57.9991091
28:4	EUFA	44.0573009	55.9426991
30:2	EUFA	37.4096859	62.5903141
13:1	OUFA	70.1842538	29.8157462
15:1	OUFA	57.9529789	42.0470211
15:2	OUFA	64.0332221	35.9667779
17:1	OUFA	54.6421829	45.3578171
17:2	OUFA	33.3479379	66.6520621
17:3	OUFA	45.4672314	54.5327686
19:1	OUFA	53.9502363	46.0497637
19:2	OUFA	49.1864247	50.8135753
19:3	OUFA	50.2714476	49.7285524
19:4	OUFA	37.0925232	62.9074768
21:1	OUFA	67.0719442	32.9280558
21:2	OUFA	52.1173867	47.8826133
21:4	OUFA	39.4096071	60.5903929
23:1	OUFA	65.9561378	34.0438622
25:1	OUFA	61.8598981	38.1401019
27:1	OUFA	55.8013164	44.1986836
27:3	OUFA	73.3677863	26.6322137
29:3	OUFA	72.8375788	27.1624212
20:5	PUFA	52.7460392	47.2539608
21:5	PUFA	49.8376263	50.1623737
22:5	PUFA	51.2505819	48.7494181
22:6	PUFA	53.4051001	46.5948999
23:5	PUFA	62.4345273	37.5654727
24:5	PUFA	57.9844112	42.0155888
24:6	PUFA	54.2806776	45.7193224
26:5	PUFA	49.1759863	50.8240137
26:6	PUFA	62.8657577	37.1342423
28:5	PUFA	47.3825059	52.6174941
28:6	PUFA	65.2910235	34.7089765
30:5	PUFA	73.6114813	26.3885187
30:6	PUFA	83.0481549	16.9518451

Abbreviations: HSM, human population from Moscow; HSS, human population from Shanghai; SFA, short-chain fatty acids; EUFA, even-chain unsaturated fatty acids; OUFA, odd-chain unsaturated fatty acids; PUFA, polyunsaturated fatty acids.

Supplementary Table 7. Proportion of FAs in brain samples (prefrontal cortex) across all species.

FA	FA.group	HS	PT	MM	CH	SS
14:0	SFA	18.2421545	21.17796	19.4700807	22.5424928	18.5673121
15:0	SFA	18.9108723	19.1305375	21.7076671	19.2397876	21.0111354
16:0	SFA	20.2096401	20.9223635	20.1160169	18.5360724	20.2159071
18:0	SFA	21.8385952	19.2728365	20.0910542	19.4723558	19.3251584
24:0	SFA	21.9803683	14.61936	18.9355341	18.7759591	25.6887785
26:0	SFA	27.8775837	19.872943	20.2998243	14.4133769	17.5362721
28:0	SFA	24.909516	20.0315222	23.998976	11.613011	19.4469748
16:1	EUFA	19.198328	21.4443145	20.4641566	16.7929815	22.1002195
16:2	EUFA	19.9239587	32.5149424	20.1704209	6.82673805	20.56394
16:3	EUFA	23.3654628	28.1171254	27.9941491	9.56336488	10.9598979
18:1	EUFA	20.2128312	18.4326293	19.1758533	21.7664861	20.4122002
18:2	EUFA	18.0208491	17.4342399	22.0781043	25.4884879	16.9783187
18:3	EUFA	19.7066075	22.0032536	29.9692216	13.3426002	14.978317
20:1	EUFA	18.9749921	11.914382	16.3713251	30.3456564	22.3936444
20:2	EUFA	24.4394868	19.7521618	22.6068061	15.7214414	17.4801039
20:3	EUFA	20.2131484	17.3881313	18.2360373	18.7020424	25.4606407
20:4	EUFA	20.9881347	21.0003243	21.0522227	15.8846158	21.0747025
20:5	EUFA	20.1415357	36.5639876	24.1497066	9.62230047	9.52246968
22:3	EUFA	26.9713022	17.3228207	18.2256554	17.8634705	19.6167512
22:4	EUFA	25.3350415	23.2988551	21.1130364	16.3246535	13.9284135
22:5	EUFA	18.9540404	16.4162988	17.6731354	21.4692705	25.4872549
22:6	EUFA	15.6018374	18.6516946	19.3623942	26.8632699	19.5208039
24:1	EUFA	22.3334308	21.4187979	12.5949062	22.7452046	20.9076606
24:4	EUFA	21.1887953	19.5677354	19.8305279	21.1144026	18.2985388
24:5	EUFA	18.0435436	22.1811894	18.1018709	18.9230629	22.7503331
24:6	EUFA	18.5212226	19.0647611	18.7810111	24.4361602	19.196845
17:1	OUFA	11.7904975	15.7014481	16.4484704	35.3707378	20.6888462
19:3	OUFA	10.1204188	16.576835	20.4955928	28.8767948	23.9303587
21:3	OUFA	11.5516648	13.2542954	13.6282217	43.5278333	18.0379848
26:1	LUFA	36.5753301	9.74948937	12.292236	20.0774293	21.3055152
27:1	LUFA	27.5643479	17.1841403	15.1687936	17.9914347	22.0912835
28:1	LUFA	42.8655349	12.9961609	10.448513	18.8419689	14.8478223
28:2	LUFA	42.1201366	10.969835	11.9692881	26.0201759	8.92056433

Abbreviations: HS, *Homo sapiens* ; PT, *Pan troglodytes* ; MM, *Macaca mulatta* ; CH, *Capra hircus* ; SS, *Sus scrofa* ; SFA, short-chain fatty acids; EUFA, even-chain unsaturated fatty acids; OUFA, odd-chain unsaturated fatty acids; LUFA, long-chain unsaturated fatty acids.

Supplementary Table 8. Proportion of FAs in brain samples (prefrontal cortex) across primate species.

FA	FA.group	HS	PT	MM
14:0	SFA	30.9765563	35.9617759	33.0616678
15:0	SFA	31.6504844	32.0181306	36.3313849
16:0	SFA	32.9963971	34.1600648	32.843538
18:0	SFA	35.6825297	31.4902838	32.8271865
24:0	SFA	39.5791203	26.3244637	34.096416
26:0	SFA	40.9661129	29.2032925	29.8305946
28:0	SFA	36.1321597	29.0564521	34.8113882
16:1	EUFA	31.4176626	35.0931726	33.4891648
16:2	EUFA	27.4399459	44.7806722	27.779382
16:3	EUFA	29.3991218	35.3778053	35.2230728
18:1	EUFA	34.9574056	31.8786068	33.1639875
18:2	EUFA	31.3225254	30.3029242	38.3745504
18:3	EUFA	27.4928288	30.6968962	41.810275
20:1	EUFA	40.149622	25.2099148	34.6404632
20:2	EUFA	36.5869045	29.5697886	33.8433069
20:3	EUFA	36.200071	31.1406999	32.6592291
20:4	EUFA	33.2930009	33.312337	33.3946621
20:5	EUFA	24.9106158	45.2215493	29.8678349
22:3	EUFA	43.1404316	27.7077449	29.1518235
22:4	EUFA	36.3242374	33.4048453	30.2709173
22:5	EUFA	35.7330295	30.9487622	33.3182083
22:6	EUFA	29.0992593	34.7876012	36.1131395
24:1	EUFA	39.6354328	38.0122218	22.3523454
24:4	EUFA	34.9724773	32.2968896	32.7306331
24:5	EUFA	30.9353578	38.0292832	31.035359
24:6	EUFA	32.8582758	33.8225607	33.3191634
17:1	OUFA	26.8329219	35.7334989	37.4335792
19:3	OUFA	21.4448153	35.1257366	43.4294481
21:3	OUFA	30.0557062	34.4856966	35.4585972
26:1	LUFA	62.3970784	16.632513	20.9704086
27:1	LUFA	46.0040026	28.6797728	25.3162246
28:1	LUFA	64.6439449	19.5990349	15.7570202
28:2	LUFA	64.7411864	16.861297	18.3975166

Abbreviations: HS, Homo sapiens; PT, Pan troglodytes; MM, Macaca mulatta; SFA, short-chain fatty acids; EUFA, even-chain unsaturated fatty acids; OUFA, odd-chain unsaturated fatty acids; LUFA, long-chain unsaturated fatty acids.

Supplementary Table 9. Proportion of FAs in brain samples (cerebellum) across all species.

FA	FA.group	HS	PT	MM	CH	SS
14:0	SFA	28.5318135	19.2233036	17.2162707	15.7281969	19.3004153
15:0	SFA	23.6259077	13.8043198	22.5235852	19.0349029	21.0112844
16:0	SFA	22.2362953	20.9613166	21.3504669	17.7802584	17.6716628
18:0	SFA	21.9051667	21.2652264	20.9214767	18.1995054	17.7086249
24:0	SFA	18.2135085	12.1034279	20.6513091	19.8489239	29.1828305
26:0	SFA	24.6720667	19.7020715	17.3678056	12.6744689	25.5835872
28:0	SFA	14.8843362	14.8675341	17.5819568	8.69872893	43.9674439
16:1	EUFA	25.2763967	31.0139795	18.4070115	10.0885098	15.2141026
16:2	EUFA	22.5523441	38.7860966	12.3755806	7.88760093	18.3983777
16:3	EUFA	27.1387683	30.2993249	21.1028538	9.56087258	11.8981805
18:1	EUFA	20.4576827	19.3421024	20.2649996	20.1327825	19.8024328
18:2	EUFA	24.5776781	16.0050171	29.5523381	16.5007823	13.3641844
18:3	EUFA	17.6822756	15.1081413	33.6104813	11.0534905	22.5456113
20:1	EUFA	15.2169098	14.9208749	17.1257104	36.3382581	16.3982469
20:2	EUFA	20.101875	17.1014708	22.2496809	21.5111476	19.0358256
20:3	EUFA	13.7642683	19.316505	12.8735453	18.7081971	35.3374843
20:4	EUFA	21.2700632	20.9213879	21.0913693	15.5854364	21.1317432
20:5	EUFA	24.3468343	45.3224388	18.9955027	5.74913919	5.58608511
22:3	EUFA	14.7557564	25.1418531	15.3145412	20.5471733	24.2406759
22:4	EUFA	21.8739599	23.7903347	18.9668392	19.9679288	15.4009373
22:5	EUFA	13.3115693	12.9839675	15.7141801	25.3027789	32.6875042
22:6	EUFA	17.1066625	16.5411214	19.8688167	26.6900815	19.7933179
24:1	EUFA	16.0298767	19.3808762	18.1390065	24.521933	21.9283075
24:4	EUFA	18.2187627	17.0782325	24.0868416	20.3058953	20.3102678
24:5	EUFA	15.7160898	17.5258698	19.255817	28.1344229	19.3678005
24:6	EUFA	11.6170698	17.3982205	26.3320622	21.6932288	22.9594187
17:1	OUFA	15.2227143	13.6085611	17.2373541	32.166647	21.7647235
19:3	OUFA	8.45493667	14.2290374	12.1699836	35.0142301	30.1318122
21:3	OUFA	9.80975425	12.9268527	10.9521061	47.5061234	18.8051636
26:1	LUFA	32.3929654	21.4668824	15.9265153	17.0311005	13.1825364
27:1	LUFA	32.2283596	18.5402938	14.6904904	19.4740442	15.066812
28:1	LUFA	42.2363313	29.1736329	10.2699334	12.1974321	6.12267027
28:2	LUFA	32.1044338	24.2801589	14.3965574	23.596979	5.62187082

Abbreviations: HS, Homo sapiens; PT, Pan troglodytes; MM, Macaca mulatta; CH, Capra hircus; SS, Sus scrofa; SFA, short-chain fatty acids; EUFA, even-chain unsaturated fatty acids; OUFA, odd-chain unsaturated fatty acids; LUFA, long-chain unsaturated fatty acids.

Supplementary Table 10. Proportion of FAs in brain samples (cerebellum) across primate species.

FA	FA.group	HS	PT	MM
14:0	SFA	43.9144282	29.5873372	26.4982345
15:0	SFA	39.4068478	23.0249239	37.5682283
16:0	SFA	34.4491978	32.473959	33.0768433
18:0	SFA	34.177762	33.1792885	32.6429495
24:0	SFA	35.7350118	23.7469973	40.517991
26:0	SFA	39.9599772	31.9103518	28.1296709
28:0	SFA	31.4454527	31.4099556	37.1445917
16:1	EUFA	33.8383945	41.5194968	24.6421087
16:2	EUFA	30.594375	52.6169864	16.7886386
16:3	EUFA	34.5536556	38.5777433	26.8686011
18:1	EUFA	34.0593624	32.2020673	33.7385703
18:2	EUFA	35.0433683	22.8202888	42.1363429
18:3	EUFA	26.6295729	22.7529171	50.61751
20:1	EUFA	32.1959046	31.5695547	36.2345407
20:2	EUFA	33.8113568	28.7646765	37.4239667
20:3	EUFA	29.952067	42.0341451	28.0137878
20:4	EUFA	33.6111176	33.0601382	33.3287442
20:5	EUFA	27.4594213	51.1166226	21.4239561
22:3	EUFA	26.7255599	45.5368116	27.7376284
22:4	EUFA	33.8443079	36.8094033	29.3462888
22:5	EUFA	31.6868817	30.9070578	37.4060605
22:6	EUFA	31.9651516	30.9083932	37.1264551
24:1	EUFA	29.9345447	36.1922751	33.8731802
24:4	EUFA	30.6796659	28.7590586	40.5612754
24:5	EUFA	29.936677	33.3840229	36.6793
24:6	EUFA	20.9893866	31.4346029	47.5760104
17:1	OUFA	33.043558	29.5397568	37.4166852
19:3	OUFA	24.2581825	40.8247395	34.917078
21:3	OUFA	29.1188157	38.3714648	32.5097196
26:1	LUFA	46.4173285	30.7608556	22.8218159
27:1	LUFA	49.2343128	28.3234591	22.4422281
28:1	LUFA	51.7095792	35.7170293	12.5733916
28:2	LUFA	45.3573215	34.3031427	20.3395358

Abbreviations: HS, Homo sapiens; PT, Pan troglodytes; MM, Macaca mulatta; SFA, short-chain fatty acids; EUFA, even-chain unsaturated fatty acids; OUFA, odd-chain unsaturated fatty acids; LUFA, long-chain unsaturated fatty acids.

Supplementary Table 11. Slope of the FAs change in brain samples (prefrontal cortex) across primate spe

FA	HS	PT	MM
14:0	-0.0631205	0.05761317	-0.031387
15:0	0.07709915	0.1374985	0.07995039
16:0	-0.0198	-0.0087383	0.00910073
16:1	-0.0458916	0.02792628	-0.0424941
16:2	-0.2716095	-0.1373701	-0.1227745
16:3	-0.2834764	0.01062007	-0.1185105
17:1	0.00210029	0.04919066	0.02019109
18:0	0.02787891	0.03511639	0.01902807
18:1	0.02781255	0.01014242	0.01967489
18:2	0.16097336	0.24988306	0.13946962
18:3	-0.0380624	0.16215323	0.01824495
19:3	-0.1645356	-0.0198253	-0.0876225
20:1	0.09553404	0.06180023	0.17944773
20:2	0.0932008	0.06641184	0.09819966
20:3	-0.0743783	-0.0249436	-0.0560018
20:4	-0.0073033	0	-0.0093838
20:5	-0.0623713	0.01426597	0.00086678
21:3	-0.075538	-0.0733445	-0.0184107
22:3	-0.1270709	-0.1617646	-0.1238236
22:4	0.05738793	0.02294994	-0.0116041
22:5	-0.0177963	0.03224612	0.01746873
22:6	-0.0317179	-0.0164798	0.01416885
24:0	0.01820411	0.07157941	0.06647578
24:1	0.15207405	0.0881659	0.19465434
24:4	0.08668248	0.03148932	0.05695224
24:5	-0.1938409	-0.1257519	-0.1146224
24:6	-0.0611344	-0.0163598	0.0550616
26:0	0.02534038	0.14242888	-0.026912
26:1	0.59799205	0.28480296	0.49078458
27:1	0.15919639	-0.0117898	0.01029125
28:0	-0.1729413	0.00787881	-0.139722
28:1	0.5728395	0.2066839	0.26986591
28:2	0.41703147	0.33016856	0.10144381

Abbreviations: HS, Homo sapiens; PT, Pan troglodytes; MM, Macaca mulatta.

Supplementary Table 12. Slope of the FAs change in brain samples (cerebellum) across primate species.

FA	HS	PT	MM
14:0	-0.0435892	-0.0638202	-0.1104082
15:0	0.01413199	-0.0554658	0.03867063
16:0	0.0181288	5.12e-16	-0.0145423
16:1	-0.1640054	-0.0660759	-0.2178243
16:2	-0.2979511	0.00168876	-0.3298545
16:3	-0.2483624	-0.1826051	-0.269675
17:1	0.03145842	-0.0750335	0.04224822
18:0	-0.003771	-0.0219973	-0.0140263
18:1	0.01305289	0.02973811	-0.0339442
18:2	0.12358867	0.08005423	0.13088557
18:3	-0.0010153	-0.0261401	-0.0596452
19:3	-0.0748543	-0.0489357	-0.1482564
20:1	0.11217534	-0.0016186	0.18423412
20:2	-0.0505165	0.02939267	0.03395933
20:3	-0.0485716	-0.0257825	-0.0916227
20:4	0.002842	0	-0.0088667
20:5	-0.1473175	-0.226973	-0.0202074
21:3	-0.0032949	-0.0310985	0.02976028
22:3	-0.0573095	0.12441951	-0.1481382
22:4	0.01114498	0.03415631	0.03916227
22:5	0.03286841	-0.0610769	0.03433088
22:6	-0.0403466	-0.0638944	0.03916398
24:0	-0.0230909	-0.0101038	0.09064039
24:1	-0.0165707	0.06930135	0.06672158
24:4	0.0928119	0.10392639	0.05311563
24:5	-0.1069192	-0.1233656	-0.1158986
24:6	-0.0904263	-0.1107379	0.09318646
26:0	0.00653715	0.0475627	0.08574394
26:1	0.2730178	0.34025204	0.29349114
27:1	0.22558066	0.10694694	0.23542363
28:0	-0.1160023	-0.1242435	0.03345134
28:1	0.33941246	0.13224757	0.09440133
28:2	0.27785879	0.21928188	0.21533389

Abbreviations: HS, Homo sapiens; PT, Pan troglodytes; MM, Macaca mulatta.

Supplementary Table 13. Correlation between the changes in milk and in brain

Brain regions	Milk_species	Brain_species	Coefficient	P.value
PFC	milk.HS	PFC.HS	0.746364987	1.43E-06
PFC	milk.HSm	PFC.HS	0.743225883	1.67E-06
PFC	milk.HSs	PFC.HS	0.746841658	1.40E-06
PFC	milk.MM	PFC.MM	0.807300152	4.08E-08
PFC	milk.MF	PFC.MM	0.782022993	2.04E-07
PFC	milk.CH	PFC.CH	0.474693422	6.97E-03
PFC	milk.SS	PFC.SS	0.677095271	2.88E-05
CB	milk.HS	CB.HS	0.74120384	1.85E-06
CB	milk.HSm	CB.HS	0.733121154	2.72E-06
CB	milk.HSs	CB.HS	0.747039713	1.38E-06
CB	milk.MM	CB.MM	0.779425733	2.38E-07
CB	milk.MF	CB.MM	0.749720415	1.21E-06
CB	milk.CH	CB.CH	0.393924963	2.83E-02
CB	milk.SS	CB.SS	0.669186242	3.85E-05

Abbreviations: HS, Homo sapiens; MM, Macaca mulatta; CH, Capra hircus; SS, Sus scrofa; PFC, prefrontal cortex; CB, cerebellum.

Supplementary Table 14. List of annotated fatty acids in milk.

Fatty Acid	Formula	Measured m/z	RT	Neutral Mass	Theoretical $[M–H]^-$ m/z	Mass Error (ppm)
6:0	C6H12O2	115.0748	1.03	116.0837296	115.0764532	-14.36574533
8:0	C8H16O2	143.1063	1.76	144.1150298	143.1077533	-10.15520059
10:0	C10H20O2	171.1377	2.97	172.1463299	171.1390534	-7.908287635
10:1	C10H18O2	169.1221	2.24	170.1306798	169.1234034	-7.7065171
11:0	C11H22O2	185.1535	3.59	186.1619799	185.1547035	-6.499869014
12:0	C12H24O2	199.1692	4.16	200.17763	199.1703535	-5.791754443
12:1	C12H22O2	197.1536	3.46	198.1619799	197.1547035	-5.597032688
12:2	C12H20O2	195.1376	1.95	196.1463299	195.1390534	-7.44810859
13:0	C13H26O2	213.1851	4.69	214.1932801	213.1860036	-4.238600211
13:1	C13H24O2	211.1695	4.06	212.17763	211.1703535	-4.041977321
14:0	C14H28O2	227.2008	5.23	228.2089301	227.2016537	-3.757343691
14:1	C14H26O2	225.1852	4.44	226.1932801	225.1860036	-3.568650925
14:2	C14H24O2	223.1695	3.93	224.17763	223.1703535	-3.824637845
15:0	C15H30O2	241.2167	5.88	242.2245802	241.2173037	-2.502884953
15:1	C15H28O2	239.2008	5.2	240.2089301	239.2016537	-3.568849491
15:2	C15H26O2	237.1853	4.53	238.1932801	237.1860036	-2.9664914
16:0	C16H32O2	255.2325	6.68	256.2402303	255.2329538	-1.77799776
16:1	C16H30O2	253.2168	5.48	254.2245802	253.2173037	-1.9893552
16:2	C16H28O2	251.2012	4.79	252.2089301	251.2016537	-1.806017967
16:3	C16H26O2	249.1855	4.38	250.1932801	249.1860036	-2.021021376
17:0	C17H34O2	269.2484	7.76	270.2558803	269.2486039	-0.75717414
17:1	C17H32O2	267.2327	6.18	268.2402303	267.2329538	-0.949746715
17:2	C17H30O2	265.2169	5.25	266.2245802	265.2173037	-1.522295696
17:3	C17H28O2	263.2014	4.63	264.2089301	263.2016537	-0.963803595
18:0	C18H36O2	283.2639	8.97	284.2715304	283.2642539	-1.249478305
18:1	C18H34O2	281.2482	6.96	282.2558803	281.2486039	-1.435982524
18:2	C18H32O2	279.2325	5.8	280.2402303	279.2329538	-1.625179313
18:3	C18H30O2	277.2169	5.08	278.2245802	277.2173037	-1.456399563
18:4	C18H28O2	275.2015	4.58	276.2089301	275.2016537	-0.558407618
19:0	C19H38O2	297.2794	10.52	298.2871805	297.279904	-1.695361823
19:1	C19H36O2	295.2638	8.1	296.2715304	295.2642539	-1.537377227
19:2	C19H34O2	293.2483	6.56	294.2558803	293.2486039	-1.03621322
19:3	C19H32O2	291.2326	5.93	292.2402303	291.2329538	-1.21484748
19:4	C19H30O2	289.2167	5.09	290.2245802	289.2173037	-2.087493218
20:0	C20H40O2	311.2953	12.26	312.3028305	311.2955541	-0.81614227
20:1	C20H38O2	309.2796	9.29	310.2871805	309.279904	-0.982918696
20:2	C20H36O2	307.2639	7.44	308.2715304	307.2642539	-1.151883226
20:3	C20H34O2	305.2482	6.28	306.2558803	305.2486039	-1.323079205
20:4	C20H32O2	303.2326	5.61	304.2402303	303.2329538	-1.16677167
20:5	C20H30O2	301.2169	4.95	302.2245802	301.2173037	-1.340358455

21:0	C21H42O2	325.3108	13.88	326.3184806	325.3112041	-1.242274828
21:1	C21H40O2	323.2953	10.95	324.3028305	323.2955541	-0.785848914
21:2	C21H38O2	321.2795	8.6	322.2871805	321.279904	-1.257461158
21:4	C21H34O2	317.2483	6.14	318.2558803	317.2486039	-0.957823222
21:5	C21H32O2	315.2328	5.34	316.2402303	315.2329538	-0.487904637
22:0	C22H44O2	339.3263	14.85	340.3341307	339.3268542	-1.633205192
22:1	C22H42O2	337.3109	12.47	338.3184806	337.3112041	-0.901618198
22:2	C22H40O2	335.2953	9.87	336.3028305	335.2955541	-0.757723915
22:3	C22H38O2	333.2796	8.01	334.2871805	333.279904	-0.912137205
22:4	C22H36O2	331.2639	6.77	332.2715304	331.2642539	-1.068429617
22:5	C22H34O2	329.2482	5.84	330.2558803	329.2486039	-1.226635665
22:6	C22H32O2	327.2326	5.35	328.2402303	327.2329538	-1.081198015
23:0	C23H46O2	353.3422	15.52	354.3497807	353.3425043	-0.861076254
23:1	C23H44O2	351.3265	13.95	352.3341307	351.3268542	-1.008150603
23:5	C23H36O2	343.2638	6.45	344.2715304	343.2642539	-1.322399681
24:0	C24H48O2	367.3577	16.06	368.3654308	367.3581543	-1.236720336
24:1	C24H46O2	365.3419	14.85	366.3497807	365.3425043	-1.653940708
24:2	C24H44O2	363.3266	13.16	364.3341307	363.3268542	-0.699619026
24:3	C24H42O2	361.311	10.44	362.3184806	361.3112041	-0.564958733
24:4	C24H40O2	359.2953	8.64	360.3028305	359.2955541	-0.707109946
24:5	C24H38O2	357.2795	7.25	358.2871805	357.279904	-1.130757693
24:6	C24H36O2	355.2639	6.32	356.2715304	355.2642539	-0.996251484
25:0	C25H50O2	381.3735	16.56	382.3810809	381.3738044	-0.798124456
25:1	C25H48O2	379.3578	15.5	380.3654308	379.3581543	-0.933996794
26:0	C26H52O2	395.389	17.11	396.3967309	395.3894544	-1.149368591
26:1	C26H50O2	393.3736	16	394.3810809	393.3738044	-0.51956627
26:2	C26H48O2	391.3579	15.09	392.3654308	391.3581543	-0.649837744
26:4	C26H44O2	387.3267	11.3	388.3341307	387.3268542	-0.398088535
26:5	C26H42O2	385.3109	9.41	386.3184806	385.3112041	-0.789299446
26:6	C26H40O2	383.2952	7.87	384.3028305	383.2955541	-0.923729629
27:1	C27H52O2	407.3891	16.49	408.3967309	407.3894544	-0.870047607
27:3	C27H48O2	403.3577	14.79	404.3654308	403.3581543	-1.126342173
28:1	C28H54O2	421.4048	17.01	422.412381	421.4051045	-0.722612699
28:2	C28H52O2	419.3892	16.12	420.3967309	419.3894544	-0.606711059
28:4	C28H48O2	415.3579	14.14	416.3654308	415.3581543	-0.612289171
28:5	C28H46O2	413.3421	12.27	414.3497807	413.3425043	-0.978014203
28:6	C28H44O2	411.3263	10.11	412.3341307	411.3268542	-1.347323605
29:3	C29H52O2	431.389	15.78	432.3967309	431.3894544	-1.053452316
30:2	C30H56O2	447.4202	17.08	448.428031	447.4207546	-1.239498021
30:5	C30H50O2	441.3732	14.63	442.3810809	441.3738044	-1.369324038
30:6	C30H48O2	439.3577	13.11	440.3654308	439.3581543	-1.034052277

Supplementary Table 15. List of annotated fatty acids in brain**.**

Fatty Acid	Formula	Measured m/z	RT	Neutral Mass	Theoretical [M–H]⁻ m/z	Mass Error (ppm)
14:0	C14H28O2	227.2015	5.26	228.20893	227.2016537	-0.6763802
15:0	C15H30O2	241.2176	5.94	242.22458	241.2173037	1.228190496
16:3	C16H26O2	249.1862	4.38	250.19328	249.1860036	0.788125325
16:2	C16H28O2	251.2019	5.03	252.20893	251.2016537	0.980587892
16:1	C16H30O2	253.2174	5.57	254.22458	253.2173037	0.380150956
16:0	C16H32O2	255.2333	6.75	256.24023	255.2329538	1.356393815
17:1	C17H32O2	267.2335	6.22	268.24023	267.2329538	2.04389613
18:3	C18H30O2	277.2178	5.18	278.22458	277.2173037	1.790150879
18:2	C18H32O2	279.2335	5.87	280.24023	279.2329538	1.956059958
18:1	C18H34O2	281.2491	7.06	282.25588	281.2486039	1.76403329
18:0	C18H36O2	283.2647	9.01	284.27153	283.2642539	1.574739819
19:3	C19H32O2	291.2332	5.92	292.24023	291.2329538	0.845359005
20:5	C20H30O2	301.2179	4.97	302.22458	301.2173037	1.979503809
20:4	C20H32O2	303.2333	5.65	304.24023	303.2329538	1.141684621
20:3	C20H34O2	305.2491	6.67	306.25588	305.2486039	1.625337164
20:2	C20H36O2	307.265	7.64	308.27153	307.2642539	2.428097282
20:1	C20H38O2	309.2805	9.34	310.28718	309.279904	1.927066687
21:3	C21H36O2	319.2652	7.26	320.27153	319.2642539	2.963274116
22:6	C22H32O2	327.2334	5.37	328.24023	327.2329538	1.3635436
22:5	C22H34O2	329.2492	6.16	330.25588	329.2486039	1.810582924
22:4	C22H36O2	331.2649	6.83	332.27153	331.2642539	1.95030853
22:3	C22H38O2	333.2804	8.31	334.28718	333.279904	1.488247548
24:6	C24H36O2	355.2648	6.36	356.27153	355.2642539	1.537074147
24:5	C24H38O2	357.2807	7.49	358.28718	357.279904	2.227953465
24:4	C24H40O2	359.2967	8.69	360.30283	359.2955541	3.189403506
24:1	C24H46O2	365.3435	14.87	366.34978	365.3425043	2.725511509
24:0	C24H48O2	367.3592	16.06	368.36543	367.3581543	2.846488332
26:1	C26H50O2	393.3748	16.03	394.38108	393.3738044	2.53096721
26:0	C26H52O2	395.3902	17.12	396.39673	395.3894544	1.88561377
27:1	C27H52O2	407.3905	16.51	408.39673	407.3894544	2.566467513
28:2	C28H52O2	419.3909	16.13	420.39673	419.3894544	3.446800545
28:1	C28H54O2	421.4062	17.04	422.41238	421.4051045	2.599606147
28:0	C28H56O2	423.4225	18.49	424.42803	423.4207546	4.122194959
