# Supplementary material for: Comparative analysis of milk and brain fatty acids reveals human-specific signatures in brain development
Source: Commun Biol. 2026 Apr 22;9:631. doi: 10.1038/s42003-025-09401-0 (PMC13161393; doi:10.1038/s42003-025-09401-0)
Supplement: Supplementary file 2 — Reporting Summary [file 42003_2025_9401_MOESM2_ESM.pdf]

nature portfolio

Corresponding author(s): Aleksandra Mitina, Philipp Khaitovich

Last updated by author(s): Nov 7, 2025

Reporting Summary

Nature Portfolio wishes to improve the reproducibility of the work that we publish. This form provides structure for consistency and transparency in reporting. For further information on Nature Portfolio policies, see our Editorial Policies and the Editorial Policy Checklist.

Statistics

For all statistical analyses, confirm that the following items are present in the figure legend, table legend, main text, or Methods section.

n/a	Confirmed	
☐	☒	The exact sample size (n) for each experimental group/condition, given as a discrete number and unit of measurement
☐	☒	A statement on whether measurements were taken from distinct samples or whether the same sample was measured repeatedly
☐	☒	The statistical test(s) used AND whether they are one- or two-sided<br>*Only common tests should be described solely by name; describe more complex techniques in the Methods section.*
☐	☒	A description of all covariates tested
☐	☒	A description of any assumptions or corrections, such as tests of normality and adjustment for multiple comparisons
☐	☒	A full description of the statistical parameters including central tendency (e.g. means) or other basic estimates (e.g. regression coefficient) AND variation (e.g. standard deviation) or associated estimates of uncertainty (e.g. confidence intervals)
☐	☒	For null hypothesis testing, the test statistic (e.g. F, t, r) with confidence intervals, effect sizes, degrees of freedom and P value noted<br>*Give P values as exact values whenever suitable.*
☒	☐	For Bayesian analysis, information on the choice of priors and Markov chain Monte Carlo settings
☒	☐	For hierarchical and complex designs, identification of the appropriate level for tests and full reporting of outcomes
☐	☒	Estimates of effect sizes (e.g. Cohen's d, Pearson's r), indicating how they were calculated

Our web collection on statistics for biologists contains articles on many of the points above.

Software and code

Policy information about availability of computer code

Data collection	Thermo Scientific™ Xcalibur™ software version 3.0 was used to manage and record the mass-spectrometry data
Data analysis	ProteoWizard 3.0.18372 was used to convert raw spectra to .mxML<br>Isotopologue Parameter Optimization (IPO) version 3.5 was used for parameter optimization for peak picking<br>XCMS version 3.4.4 was used for peak picking and alignment<br>CAMERA version 1.33.3 was used for annotation of peaks generated by XCMS<br>R version 3.4.0 was used for statistical analysis

For manuscripts utilizing custom algorithms or software that are central to the research but not yet described in published literature, software must be made available to editors and reviewers. We strongly encourage code deposition in a community repository (e.g. GitHub). See the Nature Portfolio guidelines for submitting code & software for further information.

Data

Policy information about availability of data

All manuscripts must include a data availability statement. This statement should provide the following information, where applicable:

- Accession codes, unique identifiers, or web links for publicly available datasets
- A description of any restrictions on data availability
- For clinical datasets or third party data, please ensure that the statement adheres to our policy

The metabolomics data have been deposited to MetaboLights repository with the study identifier MTBLS12481.

Research involving human participants, their data, or biological material

Policy information about studies with human participants or human data. See also policy information about sex, gender (identity/presentation), and sexual orientation and race, ethnicity and racism.

Reporting on sex and gender	All human participants who consented to breast milk donation (n = 151) were biological women. To study differences in milk composition, we also use children's biological sex as reported by their mothers. Of the 568 human milk samples that we examined in this study, 276 (48.6%) were obtained from mothers who had sons, 180 (31.7%) were from mothers who had daughters, and for 112 samples (19.7%) we had no information about the sex of the child. To study differences in human brain composition, we use the biological sex as reported in the biobank. Of the 92 human brain samples, 52 (56.5%) were from women and 40 (43.5%) were from men.
Reporting on race, ethnicity, or other socially relevant groupings	In our milk analysis, we categorize our samples into two major groups: Eastern European and East Asian, corresponding to the collection locations in Moscow, Russia, and Shanghai, China, respectively. We did not assess the genetic background or require reporting of ethnic identity from the participants. We rely on the premise that the composition of milk lipids is primarily influenced by the mother's dietary choices, and dietary patterns tend to align with cultural habits, thus remaining consistent within specific geographical locations. As indicated by our analysis, population emerged as the primary factor determining differences in milk composition. Other contributing factors, in descending order, include the stage of lactation, the number of children a woman previously had, the mode of delivery (vaginal or cesarean birth), and the sex of the child.
Population characteristics	The data collected from the human participants who consented to donating milk included mother's age (mean age = 31 years in EEU, and 32 years in EAS), mother's height (mean height = 166 cm in EEU, and 165 cm in EAS), mother's weight (mean weight = 60 kg in EEU and 63 kg in EAS), number of children a woman had (50 samples from women who had their first child, 71 samples from women who had their second child, 14 samples from women who had their third child, and 6 samples from women who had their fourth child in EEU; 167 samples from woman who had their first child and 91 sample from women who had their second child in EAS), the mode of delivery (142 samples from women who had vaginal birth and 68 samples from woman who had cesarean section in EEU; 106 samples from women who had vaginal birth and 155 samples from woman who had cesarean section in EAS); biological sex of the child (please see above), height of the child at birth (mean height 51 cm in both EEU and EAS populations), weight of the child at birth (mean weight = 3,300 g in EEU, and 3,200 g in EAS). The data on the brain samples included information on participants' age (ranging from a minimum of 0 days to a maximum of 365 days) and biological sex (53 samples from female participants and 40 from male participants).
Recruitment	Participants for milk donation were enlisted through social media in Eastern European (EEU) population and medical centers in East Asian (EAS) population. This has the potential to introduce a self-recruitment bias between the two populations, particularly regarding the duration of lactation. The mean lactation duration in EEU cohort was one year at the time of participation, while in EAS cohort, it was one month. This disparity may be explained by the fact that participants in EEU were recruited via social media, allowing recruitment from delivery until weaning, which typically occurs around 2 years of the child's age in Eastern Europe. In East Asia, the majority of participants were recruited during the initial month after delivery, possibly due to mothers staying at specialized medical centers during this period. To control for the bias in lactation duration, we retained samples from mothers with matched lactation stage when comparing samples obtained from the two human populations.
Ethics oversight	The use of human samples was approved by the Institutional Research Ethics Boards (Skolkovo Institute of Science and Technology, Moscow, Russia) and written informed consent was obtained from all human participants.

Note that full information on the approval of the study protocol must also be provided in the manuscript.

Field-specific reporting

Please select the one below that is the best fit for your research. If you are not sure, read the appropriate sections before making your selection.

☒ Life sciences ☐ Behavioural & social sciences ☐ Ecological, evolutionary & environmental sciences

For a reference copy of the document with all sections, see nature.com/documents/nr-reporting-summary-flat.pdf

Life sciences study design

All studies must disclose on these points even when the disclosure is negative.

Sample size	No sample size calculation was performed The sample size was determined based on the availability of the samples.
Data exclusions	In both the milk and brain datasets, we excluded samples (n = 8 and n = 2, respectively) identified as outliers in the multidimensional scaling analysis.
Replication	Due to the substantial number of samples and the specificity of the mass-spectrometry experiment, we opted not to inject technical replicates for our samples. Given the size of the milk dataset (n = 921) and the time required for one sample acquisition (~20 min), this approach ensured an uninterrupted run lasting two weeks. Prolonging the experiment could potentially introduce bias due to factors like buffer evaporation or timing since the instrument calibration. The introduction of technical replicates would extend the acquisition time to four weeks (for two technical replicates) or six weeks (for three technical replicates), increasing the risk of potential batch effects. Consequently, we chose not to use technical replicates and instead relied on the mean intensity of fatty acids across all samples of a particular species, serving as biological replicates in our study.
Randomization	Milk samples were stratified by species, stage of lactation (days after parturition or birth of offspring), and in the case of human milk, by population, sex of the child and mode of delivery. Brain samples were stratified by species, brain region (PFC or CB), age, and biological sex when available.
Blinding	Blinding was not applicable to our study, as we did not hold any preconceived expectations regarding the lipid composition of milk or brain between the species.

Reporting for specific materials, systems and methods

We require information from authors about some types of materials, experimental systems and methods used in many studies. Here, indicate whether each material, system or method listed is relevant to your study. If you are not sure if a list item applies to your research, read the appropriate section before selecting a response.

Materials & experimental systems

n/a	Involved in the study	
☒	☐	Antibodies
☒	☐	Eukaryotic cell lines
☒	☐	Palaeontology and archaeology
☐	☒	Animals and other organisms
☒	☐	Clinical data
☒	☐	Dual use research of concern
☒	☐	Plants

Methods

n/a	Involved in the study	
☒	☐	ChIP-seq
☒	☐	Flow cytometry
☒	☐	MRI-based neuroimaging

Animals and other research organisms

Policy information about studies involving animals; ARRIVE guidelines recommended for reporting animal research, and Sex and Gender in Research

Laboratory animals	We used milk samples collected from rhesus macaque (Macaca mulatta) and crab-eating macaque (Macaca fascicularis) and brain samples collected from chimpanzee (Pan troglodytes) and rhesus macaque (Macaca mulatta). Chimpanzee brain samples (n = 18) were obtained from the Max Planck Institute for Evolutionary Anthropology in Leipzig, and rhesus macaque samples (n = 38) were obtained from the Yunnan Key Laboratory of Primate Biomedical Research, China. The age ranged from 0 to 45 days in chimpanzees and from 0 to 358 days in macaques. All non-human primates in this study experienced death unrelated to their participation in the experiment, were acquired legally and were stored and handled in accordance with national and local laws and regulations and the Institute for Laboratory Animal Research (ILAR) Guide for Care and Use of Laboratory Animals.
Wild animals	The study did not involve wild animals.<br>Instead, we used the following domestic species to collect milk samples: domestic cow (Bos taurus), domestic goat (Capra hircus), domestic pig (Sun scrofa), domestic yak (Bos Grunniens). Milk was collected by animal care staff, ensuring that the process did not cause stress to the animals. Brain samples were collected for domestic goat (Capra hircus) and domestic pig (Sun scrofa). Domestic animals were available through the private farms. Brain samples were collected as by-products from animals used for meat production (Ch and Sc) unrelated to the experiment.
Reporting on sex	For the milk analysis, only female animals (dams) were considered. Information on the offspring's sex was not consistently available. In the brain analysis, we collected information on the animals' sex whenever possible. This included 8 male and 10 female samples among the chimpanzee brain samples, 2 female and 36 male samples among the macaque brain samples, and 14 female and 12 male samples among the goat brain samples.
Field-collected samples	The study did not involved samples collected from the field.

Ethics oversight	The use of animal milk was approved by the Institutional Research Ethics Boards (Skolkovo Institute of Science and Technology, Moscow, Russia). Brain tissues for primates were acquired legally and were stored and handled in accordance with national and local laws and regulations and the Institute for Laboratory Animal Research (ILAR) Guide for Care and Use of Laboratory Animals.

Note that full information on the approval of the study protocol must also be provided in the manuscript.
